# The macroparasite fauna of cichlid fish from Nicaraguan lakes, a model system for understanding host–parasite diversification and speciation

**DOI:** 10.1038/s41598-022-07647-w

**Published:** 2022-03-10

**Authors:** Ana Santacruz, Marta Barluenga, Gerardo Pérez-Ponce de León

**Affiliations:** 1 Posgrado en Ciencias Biológicas, Universidad Nacional Autónoma de México, Mexico, Mexico; 2 Instituto de Biología, Universidad Nacional Autónoma de México (UNAM), Ap. Postal 70-153, C.P. 04510 Mexico, Mexico; 3 Department of Biodiversity and Evolutionary Biology, Museo Nacional de Ciencias Naturales, CSIC, José Gutiérrez Abascal, 2, 28006 Madrid, Spain; 4 Present Address: Escuela Nacional de Estudios Superiores Unidad Mérida, Km 4.5 Carretera Mérida-Tetiz, C.P. 97357 Ucú, Yucatán Mexico

**Keywords:** Biodiversity, Ichthyology

## Abstract

The Nicaraguan lakes represent an ideal continent-island-like setting to study the colonization patterns of both fish and their parasites. The dominant fish fauna are cichlids, particularly the Midas cichlid species complex *Amphilophus* spp., a well-studied model for recent sympatric speciation. Here, we characterized the Midas cichlid macroparasite diversity in Nicaraguan lakes. We evaluated patterns of parasite diversity across host populations. Morphological and molecular analyses were conducted, revealing a macroparasite fauna composed by 37 taxa, including platyhelminths, nematodes, copepods, branchiurans, hirudineans and oribatids. Three invasive species are reported for the first time. The Midas cichlid was infected by 22 parasite taxa, 18 shared with other cichlids. Eight taxa conformed the core parasite fauna of the Midas cichlid. The large lakes had higher parasite diversity than the smaller and isolated crater lakes, although parasite infracommunity diversity was lower. Environmental factors along with the differential distribution of intermediate hosts, the potential resistance gained by their hosts after colonization of new lakes, competitive exclusion among parasites, or the introduction of exotic fish, may determine the observed pattern of parasite heterogeneous distribution. Our study provides a ground to explore the evolutionary history of both, hosts and parasites within the context of speciation and diversification processes.

## Introduction

Parasitism is one of the most common ecological interactions in nature^1^. Parasites inform about the ecology and evolutionary history of their hosts^2^. Notwithstanding of the potential detrimental effect on their hosts, parasites display important roles in ecosystems. Parasites may exert strong selective pressures on host populations, regulating their populations in multiple ways^3^, determining the presence of other parasites^4^, modulating host behaviors^5–7^, and influencing host range expansion^8^. In this context, changes in parasite community composition and abundance might be an evolutionary driving force for their host populations^9–12^. The first step in understanding the structure of parasite communities and the role of these communities on hosts and ecosystems, is to collect comprehensive knowledge on parasite species composition. Besides, hosts and parasites may be the result of a long evolutionary history, and may play an important role on each other’s diversification processes^13^.

The Nicaraguan lakes system comprise two large tectonic lakes (the great Nicaraguan lakes, Managua and Nicaragua) and several young adjacent crater lakes, establishing a set-up analogous to a continent-island model. The fish fauna of this region is dominated by cichlids and poecilids, and the largest biomass corresponds to the Midas cichlid species complex (*Amphilophus* spp*.*)^14^. The Midas cichlid independently colonized each of the crater lakes in the last few thousand years by fish stocks from the great Nicaraguan lakes^15–17^. Therefore, the Midas cichlid is the outcome of recent adaptive radiations through sympatric and allopatric speciation^15,18–20^. The endemic species dwelling each crater lake display great intra-specific variation in color, body shape and trophic traits^21–23^, and recurrent phenotypes with evident evolutionary parallelisms across lakes^24^. However, the information about the parasite fauna of fish in this geographical area, and particularly in cichlids representing a model system, is still scarce.

There has been an intense effort in the Neotropics to document the freshwater fish parasite diversity. About 200 species of parasites have been described in neotropical cichlids^25^. However, a gap in the parasitological knowledge remains for Central American freshwater fishes^26^, including the Nicaraguan lakes. The most important contribution was published back in 1976^27^ with the description of the digenean fauna of fishes from Lake Nicaragua. Very few posterior studies reported fish parasites in some Nicaraguan streams of both the Pacific and Atlantic slopes^28–33^. In total, 71 species of metazoans have been reported in Nicaraguan freshwaters, but only 19 in the great Nicaraguan lakes^27,34^ and surrounding crater lakes^35^. Our recent investigation have already resulted in the description of two additional species of parasites from Nicaraguan crater lakes^36,37^, and more are expected to be discovered and described.

The objectives of this study were twofold: first, to assess the diversity of macroparasites in cichlid fishes of Nicaraguan lakes, the dominant fish group in the area, and second, to characterize the spatial distribution of parasite communities among host species and lakes, in the search for parasite diversity patterns and the processes that determine them. We hypothesized that the patterns of parasite distribution were heterogeneous among the lakes; associated to different fish community compositions and to different historical contingencies in each lake.

## Methods

### Fish collection and parasitological survey

Fish were collected from the two great Nicaraguan lakes, Managua and Nicaragua, and five crater lakes (Asososca León, Apoyeque, Xiloá, Masaya and Apoyo) during the months of November–December of three consecutive years (2017–2019). This period represents the end of the rainy season, and the time when fish breed, which allows for clear identification of fish to species level due to breeding coloration. Fish were caught with gill nets, anaesthetized with tricaine mesylate (MS-222) and photographed on a lateral standardized position for species identification. Fish were euthanized in cold water and immediately examined; fin clips were preserved in ethanol. Euthanized fish were screened for ecto- and endoparasites using a Leica EZ4 stereomicroscope. First, the fish external surface (skin, eyes, and mouth) was observed. Then, fish were dissected, and internal organs (gut, mesentery, hearth, muscle and gall, swim, and urinary bladders) were analyzed. All recovered parasites were rinsed in saline solution and stored in 100% ethanol. Representative specimens of each parasite taxa were fixed in nearly boiling 4% formalin for morphological analysis. Some specimens were fixed in 100% ethanol for DNA extraction. Gill arches were dissected and stored in 100% ethanol. Later, in the laboratory, the gills were screened for ectoparasites using a Leica EZ4 stereomicroscope.

### Ethics statement

The study protocol was approved by The Ministry of Natural Resources (MARENA) of Nicaragua (No. 001-012015). Methods to euthanase fish were carried out in strict accordance with current Spanish and European Union laws (ECC/566/2015 and 2016/63/UE, respectively), and by the American Veterinary Medical Association Guidelines for Euthanasia of Animals: 2020 edition (available at https://www.avma.org/sites/default/files/2020-02/Guidelines-on-Euthanasia-2020.pdf).

### Morphological characterization of parasites

All parasites were characterized morphologically to achieve their taxonomic identity. Parasites fixed in formalin were rinsed in distilled water and dehydrated in increasing ethanol concentrations (from 10 to 70%). Whole specimens of monogeneans, or partial sections of the body (anterior/posterior voucher regions of the body) were mounted in semi-permanent preparations of ammonium picrate-glycerin. In some cases, the haptor region or the male copulatory organ was enzymatically digested with proteinase K to recover only sclerotized structures, and then mounted on a Gray and Weiss solution for permanent preparations. Additionally, monogeneans, cestodes, trematodes and hirudineans were stained with Mayer’s paracarmine or Gomori’s trichrome and mounted on permanent slides using Canada balsam. The remaining parasite taxa, including nematodes, copepods, branchiurans and oribatid mites were cleared with a glycerin/alcohol solution (1/1) and mounted as semi-permanent slides. All specimens were photographed with an Olympus BX51 inverted light microscope equipped with differential interference contrast (DIC) optics. Voucher specimens of parasites were deposited in national collections housed at the Biology Institute, National Autonomous University of Mexico (UNAM), Mexico City, i.e., Colección Nacional de Helmintos (CNHE), Colección Nacional de Crustáceos (CNCR), and Colección Nacional de Ácaros (CNAC) (Supplementary Table S1). Additionally, the ultrastructure of the external surface of some individual parasites was analyzed through scanning electron microscopy (SEM). Individuals were dehydrated, critically point-dried, mounted on a strip of carbon conductive tape, coated with a thin layer of gold, and observed in a Hitachi SEM unit SU1510.

For further investigation of taxonomically problematic groups, specimens fixed in 100% ethanol were individually sequenced to corroborate their identification or to reveal potential cryptic diversity. For example, mitochondrial DNA sequences of the cytochrome oxidase subunit II (cox2) were obtained for larval stages of the nematode *Contracaecum* spp. which are difficult to distinguish morphologically. The phylogenetic position, nodal support and genetic distance were estimated for some parasite taxa to establish more robust species limits, and on some occasions to establish conspecificity. The molecular markers used were the mitochondrial cox1, cox2, and the nuclear genes 18S, and 28S, depending on the genetic library available for each parasite group (Supplementary Table S2). All generated sequences were submitted to GenBank (Supplementary Table S1).

### Data analysis

Parasite communities were described at the infracommunity level, i.e., all the parasite taxa occurring within an individual host within a locality^38^. This allowed us to compare all the parasite communities occurring in different host species in a single lake, but also to compare all the communities among the seven sampled lakes. The three sampling years were considered replicates and were pulled together; for ectoparasites, calculations only included data from two sampling years (2017 and 2018). The characterization of each infracommunity was based on the following ecological parameters: species richness, abundance, and diversity through Shannon–Wiener and Simpson diversity indices. Species richness is the number of parasite taxa harbored by an individual host. Abundance is the number of individuals of all conspecific parasite taxa sampled from each individual host^39^. The Shannon–Wiener diversity index estimates species diversity and their uncertainty; increasing values of the index reflect increasing diversity and evenness. Instead, Simpson diversity index estimates the richness and relative abundance of species^40^. These indices were calculated using the *diversity* function in the package ‘vegan’ in R^41^. To test the significance of parasite diversity among host species and among lakes, the non-parametric Kruskal–Wallis (KWT) and Wilcoxon tests were implemented using R^42^. In order to identify the most common parasites, prevalence of infection (proportion of hosts infected by a given parasite taxon) and mean intensity (mean number of parasites per infected host) were used to generate heatmaps of interactions with the R package ‘ggplot2’^43^.

We analyzed the representativeness of our sample for describing parasite species richness in this region with rarefaction and extrapolation curves using the R package ‘iNEXT’^44^. This approach employs Hill numbers (i.e., effective number of species). Hill numbers are a family of diversity indices that employ species richness and relative abundance, differing between them by an exponent *q*^45^. In our study we use the order of species richness (with *q* = 0). Individual hosts represent the sampling units, and the number of parasite detections through all the sampling units represents the incidence data. We used 100 bootstrap iterations to generate 95% confidence intervals for the rarefied and extrapolated curves. All the analysis were conducted in R^42^.

### Ethical approval

All applicable international, national, and/or institutional guidelines for the care and use of animals were followed.

### Field study

The Ministry of Natural Resources (MARENA) in Nicaragua provided collection permits (No. 001-012015) to MB.

## Results

### Macroparasite fauna

**Figure 1:**
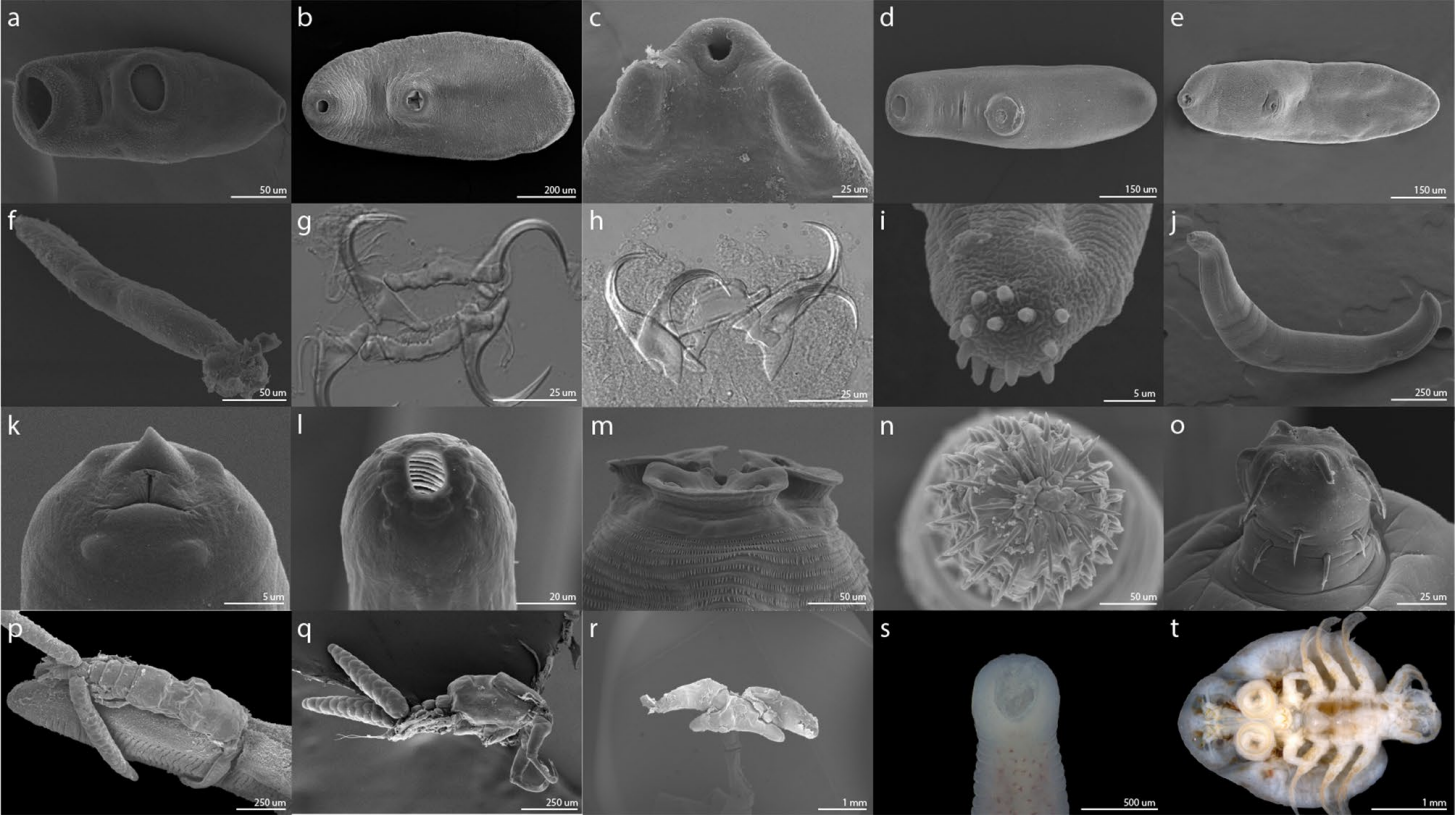
Photomicrographs of representative species of macroparasites of cichlids from the Nicaraguan lakes. The trematodes: (**a**) *Saccocoleioides* spp., (**b**) *C. cichlasomae,* (**c**) anterior end of *A. compactum*, (**d**) *O. manteri* and (**e**) metacercariae of Cryptogonomidae gen. sp. The monogeneans: (**f**) whole specimen of *S. mexicanum*, (**g**) haptor of *S. mexicanum* and (**h**) haptor of *S. nicaraguense*. The nematodes: (**i**) tail of *Physocephalus* sp., (**j,k**) larval stages of *Contracaecum* spp*.*, (**l**) apical view of *P. barlowi* and (**m**) lateral view of the anterior end of *Goezia* sp. The acanthocephalans: (**n**) apical view of the proboscis hooks of the cystacanth *Polymorphus brevis* and (**o**) lateral view of the hooks of *N. costarricense*. The copepods: (**p**) *A. margulisae* anchored to a gill filament, (**q**) Ergasilidae gen. sp. and (**r**) anchor of *L. cyprinacea*. (**s**) Oral sucker of the hirudinean *Myzobdella* sp. (**t**) Ventral view of the branchiuran *Argulus* sp.

**Figure 2:**
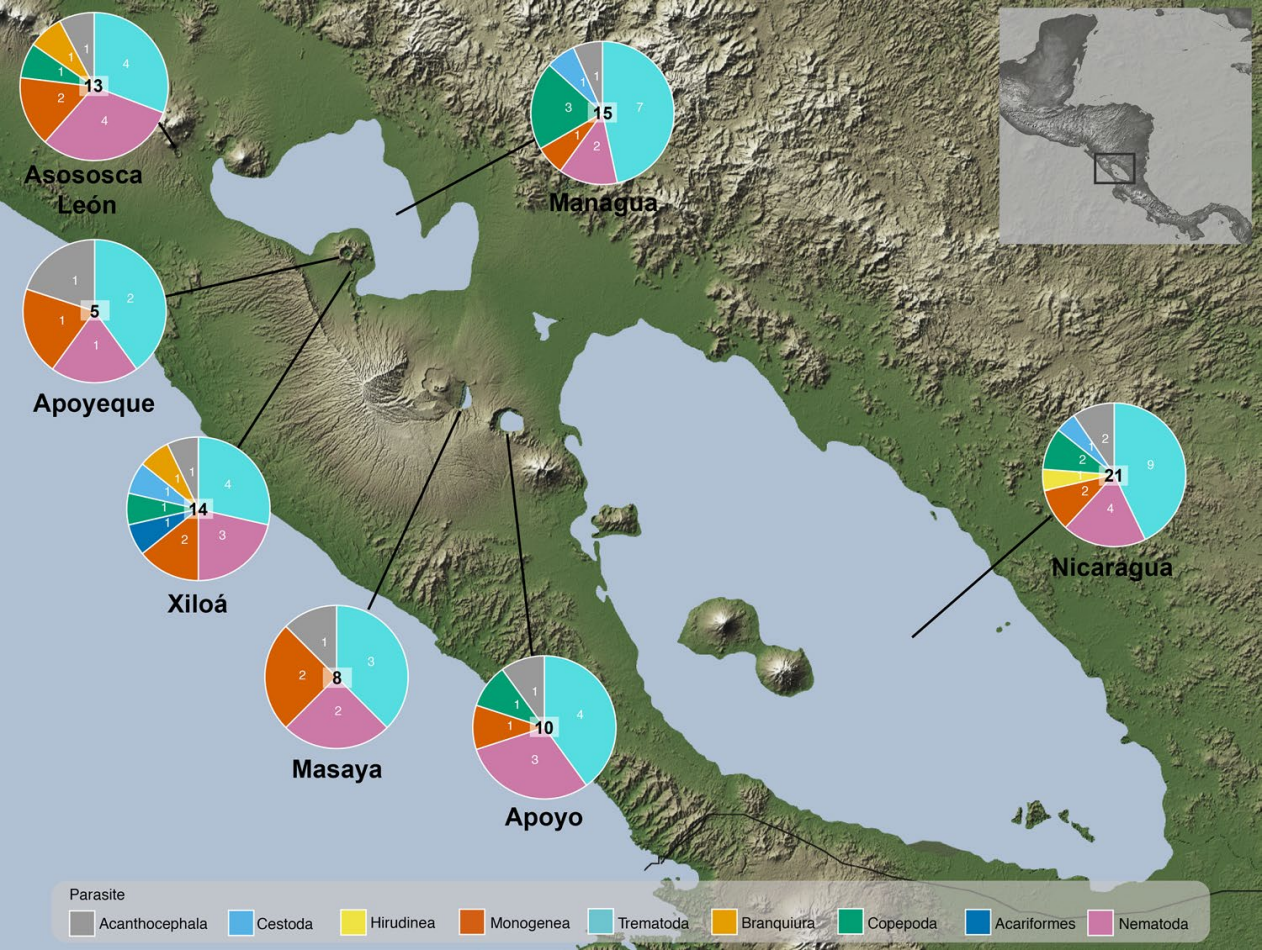
Map of the Nicaraguan lakes showing the proportion of parasite taxa per group. Numbers in the pie chart show the total number of parasite taxa per lake. Image of public domain, taken from http://photojournal.jpl.nasa.gov/catalog/PIA03364, and modified with Adobe Illustrator v. 24.2.

A total of 754 cichlid fish were studied for parasites in seven Nicaraguan lakes, belonging to 20 fish taxa, 10 of which corresponded to the Midas cichlid species complex (509 individuals) and formed the largest portion of the sample (Supplementary Table S3). Midas cichlids are particularly abundant in these lakes. Most hosts (n = 717, 94.34%) in all lakes were infected by at least one parasite taxa. We recovered over 70,000 parasites, representing 37 taxa, of which 16 were identified to species level (Fig. 1, Table 1). Considering the whole parasite community, the lakes contributed unequally to the total species richness. The great lakes Nicaragua and Managua hold the highest parasite richness, with 21 and 16 parasite taxa, respectively, and the crater lakes had lower parasite species richness, ranging from 5 to 14 taxa (Fig. 2, Supplementary Table S4). Some parasite taxa were restricted to a few lakes. Hirudineans were only found in Lake Nicaragua, oribatid mites were found in crater Lake Xiloá and cestodes were found solely in the great lakes and crater Lake Xiloá. Copepods were absent from crater Lake Apoyeque and Masaya. The remaining parasite taxa were found in all lakes.

**Table 1:** List of the macroparasite species grouped taxonomically found in cichlid fishes from the Nicaraguan lakes.

Parasite	S	T	Si	L	Midas cichlids	Other-cichlids
**Trematoda (12)**						
*Ascocotyle pindoramensis*	L	Ec	Gills	I	✓	✓
*Austrodiplostomum compactum*	L	Ec	Eye	I	✓	✓
*Crassicutis cichlasomae*	A	En	Gut	I	✓	✓
Cryptogonomidae gen. sp.	L	Ec	Gills	I	–	✓
*Ithyoclinostomum yamagutii*	L	En	Body cavity	I	–	✓
Heterophyidae gen. sp.	L	En	Body cavity	I	–	✓
*Oligogonotylus manteri*	A	En	Gut	I	✓	✓
*Posthodiplostomum* sp. 1	L	En	Body cavity	I	–	✓
*Posthodiplostomum* sp. 2	L	En	Muscle	I	–	✓
*Saccocoelioides orosiensis*	A	En	Gut	I	✓	✓
*Saccocoelioides* cf. *lamothei*	A	En	Gut	I	✓	✓
Strigeidae gen sp.	L	En	Body cavity	I	–	✓
**Nematoda (8)**						
*Contracaecum* sp. 1	L	En	Body cavity	I	✓	✓
*Contracaecum* sp. 2	L	En	Body cavity	I	✓	✓
*Contracaecum* sp. 3	L	En	Body cavity	I	✓	✓
*Goezia* sp.	A	En	Gut	I	✓	–
*Hysterothylacium* sp.	L	En	Body cavity	I	–	✓
*Physocephalus* sp.	L	En	Body cavity	I	–	✓
*Procamallanus barlowi*	A/L	En	Gut	I	✓	✓
*Rhabdochona* sp.	L	En	Gut	I	✓	–
**Monogenea (5)**						
*Sciadicleithrum mexicanum*	A	Ec	Gills	D	✓	✓
*Sciadicleithrum nicaraguense*	A	Ec	Gills	D	–	✓
*Sciadicleithrum* sp. 1	A	Ec	Gills	D	–	✓
*Sciadicleithrum* sp. 2	A	Ec	Gills	D	–	✓
Cichlidogyrus sclerosus*	A	Ec	Gills	D	–	✓
**Acanthocephala (2)**						
*Polymorphus brevis*	L	En	Body cavity	I	✓	✓
*Neoechinorhynchus costarricense*	A	En	Gut	I	✓	✓
**Cestoda (2)**						
*Cichlidocestus janikae*	A	En	Gut	I	✓	✓
Schyzocotyle acheilognathi*	A	En	Gut	I	✓	✓
**Hirudinea (1)**						
*Myzobdella* sp.	A	Ec	Skin	D	–	✓
**Copepoda (5)**						
*Acusicola margulisae*	A	Ec	Gills	D	✓	✓
*Acusicola* sp.	A	Ec	Gills	D	✓	–
Ergasilidae gen. sp.	A	Ec	Gills	D	✓	✓
Lernaea cyprinacea*	A	Ec	Skin	D	–	✓
Lernaeidae gen. sp.*	A	Ec	gIlls	D	✓	✓
**Branchiura (1)**						
*Argulus* sp.	A	Ec	Skin/mouth	D	✓	–
**Acariformes (1)**						
Oribatida gen. sp.	A	En	Body cavity	?	–	✓

*S* stage: adult A, larvae L, *T* type of parasite: Ectoparasite Ec, Endoparasite En, *Si* site of infection, *L* life cycle: direct D, indirect I, unknown ?

*Denotes invasive species.

Parasites were mainly represented by helminths, either as larval forms or as adults, because fish serve as the intermediate or definitive host (Table 1). Trematodes and nematodes were the most abundant and diverse parasite groups. Cestodes and oribatid mites were rare. Three invasive species were found, including the African monogenean *Cichlidogyrus sclerosus*, the Asian copepod *Lernaea cyprinacea*, and the Asian cestode *Schyzocotyle acheilognathi*. Twenty-two of the 37 parasite taxa were found in the Midas cichlid complex. The most common parasites in the Midas cichlid were the trematodes *Crassicutis cichlasomae*, *Oligogonotylus manteri*, and *Saccocoelioides* spp., the nematodes *Contracaecum* spp. and *Procamallanus barlowi*, the copepod Ergasilidae gen. sp., the monogenean *Sciadicleithrum mexicanum,* and the acanthocephalan *Neoechinorhynchus costarricense*, forming the Midas cichlid core parasite fauna. This core fauna was also shared with cichlids of the genus *Parachromis*. These common parasites were widely distributed in the host populations, although consistently absent from some particular crater lakes. For instance, the nematode *P. barlowi* was absent in fishes from crater Lake Xiloá, whereas the monogenean *S. mexicanum* was absent in Midas cichlids from crater lakes Asososca León and Apoyeque, but it was present in *Parachromis* spp. in both lakes.

**Figure 3:**
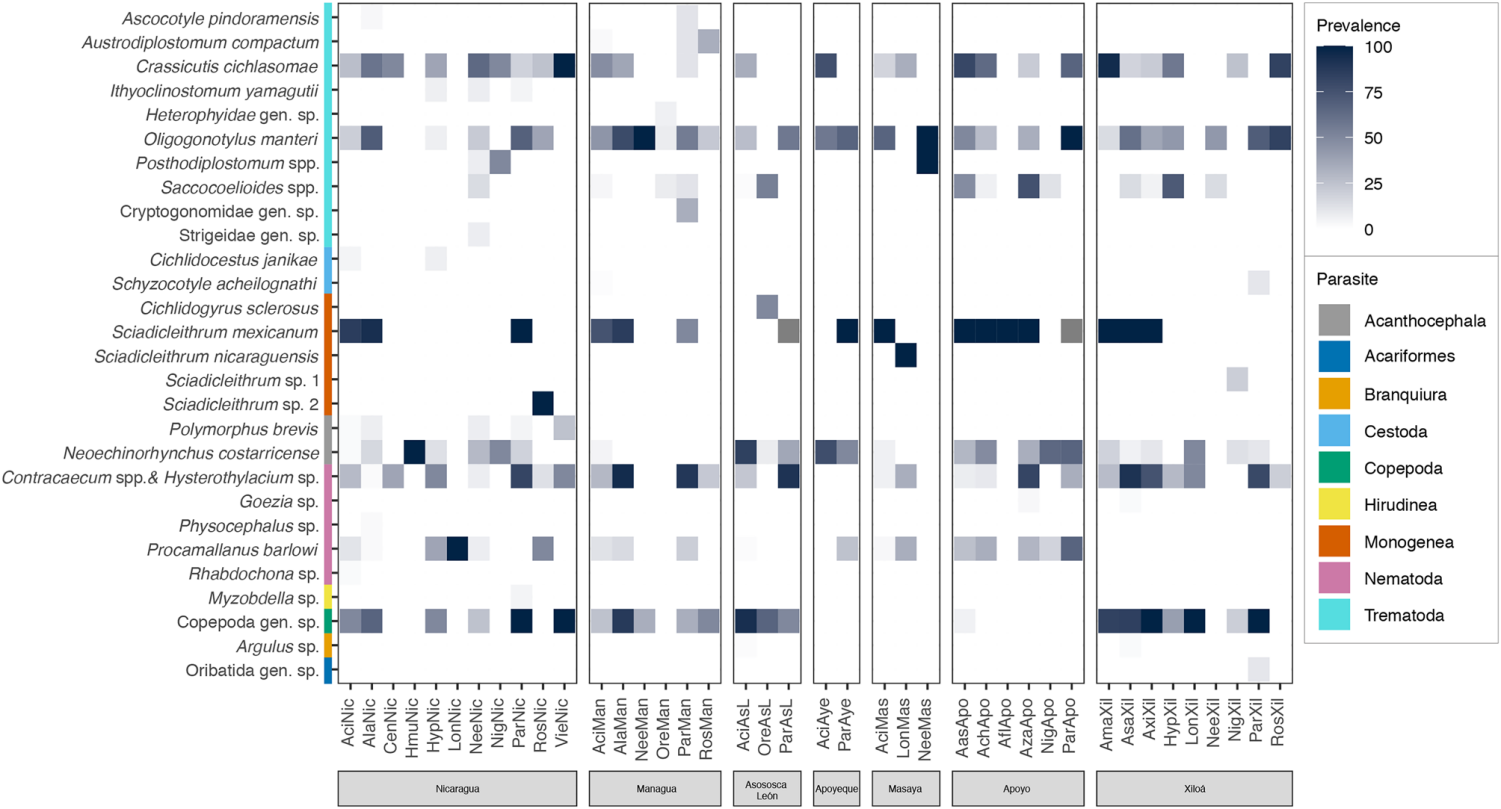
Matrix of parasite prevalence per host and lake. Dark colors represent increasing prevalence. White cells indicate absences. Host codes are according to Supplementary Table S3. (1) *Contracaecum* spp. and *Hystherothylacium* sp., (2) *S. orosiensis* and *S. cf. lamothei,* and (3) Ergasilidae copepods, were condensed each into a single taxon, and their prevalences were calculated together.

We analyzed 20 host species patchily distributed among lakes, making a total of 41 host–lake combinations. The distribution of the 37 parasite taxa was heterogeneous, with some of them present in a range between one and 29 combinations (Fig. 3). The most widely distributed parasites were the trematodes *C. cichlasomae* and *O. manteri*, and the nematodes *Contracaecum* spp. and *Hystherothylacium* sp. The heatmap depicted in Fig. 3 also showed that most parasite taxa differed largely in prevalence of infection, with some reaching values of 100%. Four parasite taxa reached infection intensities higher than 500 parasites per infected Midas cichlid, the monogenean *S. mexicanum* (up to 762 individuals) and the trematodes *Saccocoelioides* spp. (up to 1000 individuals) from crater Lake Apoyo, the heterophyd trematode (not identified to species level) (up to 1200 individuals) in *Oreochromis* sp. from Lake Managua, and the larval nematodes (up to 1801 individuals) from crater Lake Xiloá (Supplementary Fig. S1).

### Infracommunity structure among lakes and hosts

Overall, parasite species richness per individual host ranged from 0 to 8 species; richness ranged between zero and 7 and between zero and 4 for endoparasites and ectoparasites, respectively. On average, the observed richness at the infracommunity level was 2.71 species, whereas richness was 1.83 and 1.20 for endoparasites and for ectoparasites, respectively. Parasite infracommunities among lakes were significatively different in richness (KWT, p < 0.001), Shannon–Wiener (KWT, p < 0.001), and Simpson (KWT, p < 0.001) diversity indices (Table 2). Parasite infracommunities from crater Lake Apoyo were the most diverse and evenly distributed, deviating most from the rest of lakes. Parasite infracommunities reached a mean value of 3.5 parasites per individual considering all fish species, and four parasites per individual considering only the Midas cichlid. The least diverse communities where those in the extremely isolated crater Lake Apoyeque with two parasites per infected fish, and these were also evenly distributed according to the Shannon–Wiener diversity index. Interestingly, in this extremely isolated lake, no copepods were found, and the Midas cichlid populations only harbored two trematode species and one acantocephalan (see Supplementary Table S4). Crater Lake Masaya also had low infracommunity parasite diversity (2.5 parasites per fish), and copepods were not found in this lake. The large lakes had similar infracommunity richness to those of crater lakes Asososca León, Xiloá and Masaya. According to the Shannon–Wiener diversity index, crater lakes Xiloá and Masaya had the most uneven infracommunities. The Simpson diversity index revealed no major differences among lakes.

**Table 2:** Infracommunity diversity indices (Richness, Shannon–Wiener, Simpson) ± 95% confidence intervals for macroparasite taxa for each lake, and p-value of the Kruskall–Wallis or Wilcoxon tests among lakes.

Index	Great lakes		Crater lakes					
	Nicaragua^a^	Managua^b^	Asososca León^c^	Apoyeque^d^	Xiloá^e^	Masaya^f^	Apoyo^g^	p-value
Species richness	2.45 ± 0.38^g^	2.21 ± 0.43^g^	2.82 ± 0.41^dg^	2.09 ± 0.41^cg^	2.77 ± 0.35^g^	2.47 ± 0.59^g^	3.53 ± 0.39^abcdef^	p < 0.001
Shannon–Wiener	0.27 ± 0.05^g^	0.27 ± 0.05^g^	0.23 ± 0.06^g^	0.38 ± 0.15^fe^	0.14 ± 0.16^dg^	0.13 ± 0.12^dg^	0.41 ± 0.06^abce^	p < 0.001
Simpson	0.36 ± 0.05	0.39 ± 0.06^ce^	0.24 ± 0.07^b^	0.22 ± 0.09	0.21 ± 0.07^b^	0.36 ± 0.12	0.29 ± 0.06	p < 0.001

Different letters indicate statistically significant differences in paired comparisons (p-values < 0.05).

We analyzed the parasite infracommunities of the Midas cichlid species complex among and within lakes (Table 3). Infracommunity richness was largest in *A. zaliosus* from crater Lake Apoyo, and smallest in *A. citrinellus* from crater Lake Apoyeque. The Midas cichlids in crater Lake Apoyo had in general higher richness than in the rest of the lakes. Within lakes, Midas cichlid species differed in diversity indices. Within the large lakes, the lipped species *A. labiatus* consistently had larger parasite infracommunity richness and higher Shannon–Wiener index values than its congener *A. citrinellus* (Wilcoxon, L. Managua p < 0.001 and L. Nicaragua p = 0.019, respectively). Within the crater lakes, limnetic species (*A. zaliosus* in crater Lake Apoyo and *A. sagittae* in crater Lake Xiloá) had larger richness than other benthic species, although these differences were not significant (p > 0.05).

**Table 3:** Diversity indices for the infracommunities (Mean richness, observed richness, extraplolated richness, Shannon–Wiener and Simpson dominance ± 95% confidence interval).

Host-lake combination	Mean richness	Observed richness	Extrapolated richness and estimated bootstrap s.e.	Shannon–Wiener	Simpson
**Nicaragua**					
*Amphilophus citrinellus*	*2.50* ± *1.00*	*8*	*8.48* ± *1.29*	*0.12* ± *0.10*	*0.36* ± *0.11*
*Amphilophus labiatus*	*3.47* ± *0.72*	*7*	*7.00* ± *0.48*	*0.37* ± *0.10*	*0.36* ± *0.11*
*Parachromis* spp.	3.66 ± 1.84	9	11.86 ± 4.28	0.41 ± 0.14	0.35 ± 0.15
*Amatitlania nigrofasciata*	2.00 ± 2.00	3	4.50 ± 2.25	0.34 ± 0.41	0.25 ± 0.55
*Archocentrus centrarchus*	1.00 ± 0.67	2	2.00 ± 0.34	0.14 ± 0.22	0.47 ± 0.27
*Cribroheros longimanus*	1	1	1.00 ± 0.20	0	0
*Cribroheros rostratus*	3	3	4.00 ± 2.00	0.17 ± 0.21	0.21 ± 0.23
*Hypsophrys nematopus*	2.25 ± 1.05	9	16.42 ± 10.85	0.30 ± 0.17	0.25 ± 0.20
*Hypsophrys nicaraguensis*	1.87 ± 0.81	8	12.21 ± 6.76	0.40 ± 0.16	0.55 ± 0.18
*Vieja* sp.	4	4	4.18 ± 0.58	0.21 ± 0.36	0.64 ± 0.32
*p*-value	**p < 0.01**			**p < 0.05**	0.191
**Managua**					
*Amphilophus citrinellus*	*2.44* ± *0.69*	*10*	*10.00* ± *0.58*	*0.26* ± *0.07*	*0.30* ± *0.07*
*Amphilophus labiatus*	*4.20* ± *0.91*	*6*	*6.00* ± *0.21*	*0.63* ± *0.15*	*0.40* ± *0.16*
*Parachromis* spp.	2.00 ± 1.95	5	5.88 ± 1.97	0.25 ± 0.23	0.25 ± 0.23
*Cribroheros rostratus*	1.00 ± 1.03	3	3.22 ± 0.66	0.07 ± 0.24	0.72 ± 0.23
*Hypsophrys nematopus*	1	1	1.00 ± 0.28	0	0
***Oreochromis* sp.**	0.50 ± 0.66	2	2.94 ± 1.92	0	0.84 ± 0.20
*p*-value	**p < 0.001**			**p < 0.05**	0.310
**Asososca León**					
*Amphilophus citrinellus*	*3.00* ± *0.55*	*7*	*7.98* ± *2.20*	*0.29* ± *0.08*	*0.22* ± *0.09*
*Parachromis* spp.	2.28 ± 1.02	5	5.47 ± 1.27	0.18 ± 0.15	0.19 ± 0.15
***Oreochromis* sp.**	2.33 ± 1.51	4	5.85 ± 3.49	0	0.38 ± 0.19
*p*-value	0.227			0.085	0.085
**Apoyeque**					
*Amphilophus citrinellus*	*2.09* ± *0.42*	*3*	*3*	*0.39* ± *0.16*	*0.22* ± *0.10*
*Parachromis* spp.	2	5	12.50 ± 7.57	0.34 ± 0.39	0.23 ± 0.28
*p*-value	0.864			0.860	0.860
**Xiloá**					
*Amphilophus amarillo*	*2.92* ± *0.73*	*6*	*6.00* ± *0.25*	*0.21* ± *0.15*	*0.18* ± *0.16*
*Amphilophus sagittae*	*3.60* ± *0.62*	*6*	*6.00* ± *0.35*	*0.04* ± *0.11*	*0.08* ± *0.12*
*Amphilophus xiloaensis*	*3.11* ± *0.90*	*7*	*7.47* ± *1.26*	*0.11* ± *0.15*	*0.16* ± *0.16*
*Parachromis* spp.	5.50 ± 0.97	6	8.72 ± 4.03	0.07 ± 0.22	0.03 ± 0.22
*Amatitlania nigrofasciata*	1.12 ± 1.14	3	3.43 ± 1.18	0	0.62 ± 0.27
*Cribroheros longimanus*	4	3	4.00 ± 2.00	0.23 ± 0.30	0.12 ± 0.49
*Cribroheros rostratus*	2.50 ± 1.32	4	4.41 ± 1.13	0.45 ± 0.29	0.31 ± 0.34
*Hypsophrys nematopus*	0.75 ± 2.20	2	2.00 ± 0.49	0.09 ± 0.26	0.64 ± 0.28
*Hypsophrys nicaraguensis*	2.20 ± 1.47	5	5.00 ± 0.46	0.46 ± 0.24	0.29 ± 0.10
*p*-value	**p < 0.001**			0.1248	**p < 0.01**
**Masaya**					
*Amphilophus citrinellus*	*2.58* ± *0.65*	*6*	*7.93* ± *3.63*	*0.11* ± *0.13*	*0.29* ± *0.14*
*Cribroheros longimanus*	3	3	5.00 ± 2.92	0.23 ± 0.43	0.50 ± 0.46
*Hypsophrys nematopus*	3	2	2	0.69	0.5
*p*-value	0.128			0.180	0.200
**Apoyo**					
*Amphilophus astorquii*	*3.63* ± *0.68*	*7*	*7.00* ± *0.34*	*0.55* ± *0.12*	*0.33* ± *0.14*
*Amphilophus chancho*	*3.37* ± *0.69*	*7*	*7.00* ± *0.21*	*0.44* ± *0.12*	*0.37* ± *0.12*
*Amphilophus zaliosus*	*4.76* ± *0.77*	*8*	*8.00* ± *0.47*	*0.40* ± *0.11*	*0.24* ± *0.13*
*Amphilophus globosus*	*4.00* ± *1.95*	*5*	*5.33* ± *0.77*	*0.39* ± *0.14*	*0.19* ± *0.08*
*Parachromis* spp.	3.33 ± 1.77	5	5.11 ± 0.40	0.95 ± 0.40	0.55 ± 0.41
***Amatitlania nigrofasciata***	1.28 ± 1.17	3	3.00 ± 0.39	0.04 ± 0.16	0.14 ± 0.18
*p*-value	**p < 0.001**			**p < 0.001**	**p < 0.01**

*s.e*. standard error.

p-values according to Kruskall–Wallis test.

Italic values denotes the Midas cichlids.

Significance values are given in bold.

**Figure 4:**
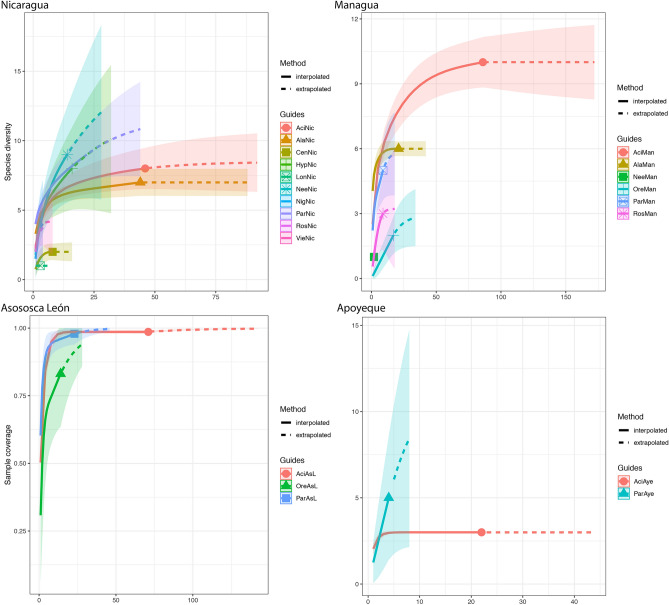

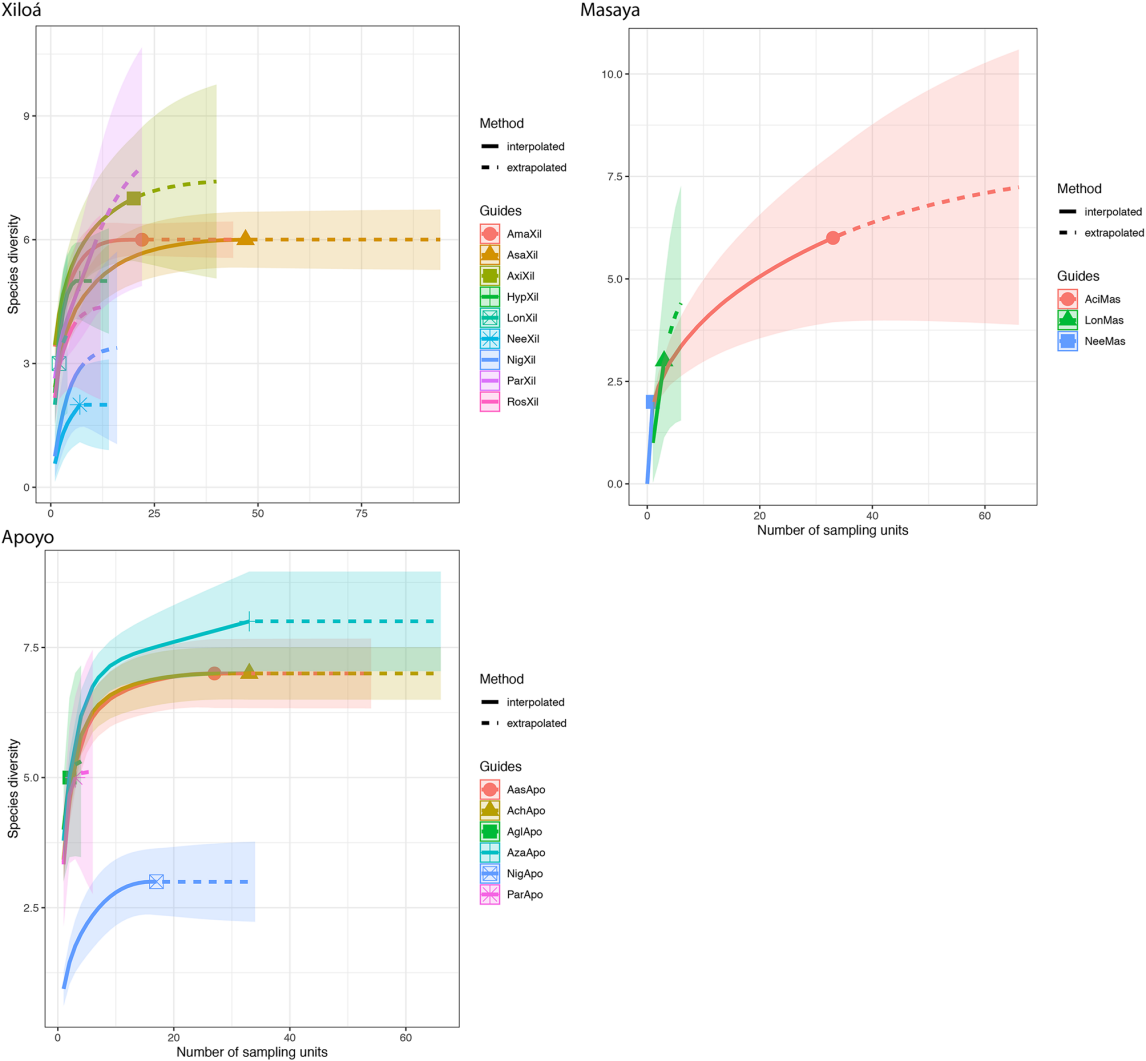
Rarefaction (solid lines) and extrapolation (dotted lines) curves for species richness (order q = 0) with 95% confidence intervals (shaded areas). The curves indicate the extrapolation of parasite richness according to the number of fish sampled.

Rarefaction and extrapolation curves for species richness indicated that sampling size was representative for the Midas cichlid complex (Fig. 4) to describe the structure of metazoan parasite infracommunities, except for some rare species in crater Lake Apoyo, and in crater Lake Masaya, where more samples from *A. citrinellus* would improve the sample. The extrapolated curves suggested that expected parasite richness was highest in the great Nicaraguan lakes, Nicaragua and Managua confirming the rest of results (Tables 2, 3).

## Discussion

Parasites represent a large fraction of the Earth’s total biodiversity^1^. It has been estimated that they account for something between one-third to over half of the species on the planet^46^. Cichlid fish are a model system in evolutionary biology due to their vast diversity and spectacular diversification rates, particularly those of the East African Great lakes^47^, but their parasite faunas still remain understudied. Cichlid parasites provide a great opportunity for exploring the role of host–parasite coevolution in adaptive radiations, and can even provide information about their biogeography and dispersal history^25^. Our study represents the first comprehensive survey of the metazoan parasite fauna of cichlids in the Nicaraguan lakes. Emphasis was put on the Midas cichlid species complex (*Amphilophus* spp*.*) because they represent a model system in evolutionary biology for their recent, repeated, and often sympatric adaptive radiations^15,18–20,23,48,49^. Here, we provide the first host-parasite records for some crater lakes and their cichlid hosts.

Our inventory of the macroparasites infecting cichlid fish in the Neotropical region of the Nicaraguan great lakes identified 37 parasite taxa in 20 cichlid taxa, and a subset of 22 in the Midas cichlid species complex. This duplicates the information existing for the lake’s region and considerably increases the information on this species complex. The most common parasites were endoparasites (60% of the parasites found), and the most common taxa were trematodes and nematodes. This agrees with what has been shown for other Neotropical cichlids^50^, but contrasts with the records reported for African cichlids, where the most common parasites found so far are ectoparasites (monogeneans), while nematodes, and particularly trematodes, are considerably less common^9–12,25,51^. Even if we are aware of geographic and taxonomic bias in the study of cichlid parasites (much greater interest on ectoparasites in Africa), the data supports a very marked historical signature of parasite communities in both African and American flocks^52^.

Previous studies have shown that each fish family possess their own set of parasites with tight taxonomical associations^50,53^, but the environment also shapes parasites communities, forming characteristic regional or biogeographic faunas^50,54,55^. Cichlids occurring in Nicaraguan freshwaters harbor a parasite community typical of the Neotropical region. The core parasite fauna of the Midas cichlid consisted of at least three trematodes, two nematodes, one acanthocephalan, one copepod, and one monogenean. These species are relatively common among cichlids occurring in Central America^28–30^, and some species, e.g. *Oligogonotylus manteri* is found in cichlids further north in southeastern Mexico^56^. Moreover, trematodes were the most diverse and common parasites and, *Crassicutis cichlasomae* was particularly abundant.

The Nicaraguan lakes form a set-up analogous to a continent-island model and, in accordance with this model, the great Nicaraguan lakes which resemble the continent, hold larger parasite diversity than in the smaller and younger crater lakes. The larger size, connections to surrounding rivers, and larger number of cichlid and non-cichlid species, might explain these differences. Interestingly, infracommunity diversity was not larger in the great lakes. On average, each fish had three parasite taxa, two of them endoparasites, and one ectoparasite. Parasite infections reached intensities as high as over 750 monogeneans, 1000 trematodes or 1800 larval nematodes in individual hosts. Occasionally, these high intensities have been previously reported in other cichlids. For the monogenean *S. mexicanum* in *Mayaheros urophthalmus*, Ref.^57^ reported mean intensity values of 223 worms per infected fish, with a range of 5–1334 parasites.

In crater Lake Masaya rarefaction and extrapolation curves suggest that sample sizes might be insufficient to adequately describe their macroparasite fauna. It is interesting to note that another very isolated crater lake (Asososca León), despite having a very impoverished fauna, possess several unique parasites that increase richness values (average of three parasites per fish). In this lake we found two nematode species, that despite intense sampling effort were not found in any other lake. These nematodes, that infect fish as larval stages, complete their life-cycle in piscivorous birds, their definitive hosts, that should easily disperse them across the region, potentially interconnecting populations of different lakes^58^. It does not seem to be the case for these parasites. The intermediate host for these nematodes (most represented by copepods) might be absent in other crater lakes. Unfortunately, no information regarding the invertebrate fauna of Nicaraguan lakes has been published, and therefore it is not possible to test the hypothesis that differences among the intensity of infection by nematodes is the result of differences in the abundance of their copepod intermediate hosts. This is also true for the potential role of snails in the life cycle of the trematodes that infect the Midas cichlid. No information is available about the diversity and abundance of these mollusks in the large and the crater lakes of Nicaragua. However, they may influence the prevalence and mean intensity of the trematodes infecting the Midas cichlid in the different lakes. Furthermore, in crater Lake Asososca León, the Midas cichlid was not infected with monogeneans and, instead, these cichlids harbored a large number of copepods on the gills. This is a very interesting finding that could be indicative of a loss of monogeneans upon colonization due to stochastic events, although the sister species *Parachromis* is infected by this parasite in this lake. Alternatively, the host might have gained resistance and/or the parasite lost infection capacity. This result could also imply competitive exclusion among parasite taxa. Experimental infection with these two ectoparasites might aid elucidating these alternatives. Interestingly, monogeneans were also absent in the Midas cichlid in crater Lake Apoyeque, although copepods were also not found in this lake. Still, some of these factors may explain the heterogenous values of infracommunities and describe why crater Lake Apoyo harbored the richer parasite infracommunities, whereas those of crater Lake Apoyeque had the poorest.

In the fish impoverished, yet parasite diverse, crater Lake Apoyo, the convict cichlid *Amatitlania nigrofasciata* was introduced from a nearby crater lake in 2018 (MB pers. obs.). The specimens of *A. nigrofasciata* from our study harbored only three parasite species, all of which are common cichlid parasites. On average, compared to populations of this species in other lakes, no significant difference of parasite infracommunity diversity was found. However, it is notable that convict cichlids in crater Lake Apoyo were infected by the nematode *P. barlowi* which is known to be abundant in native Midas cichlids from the same lake. This appears to be a case of parasite spillback and should be the subject of further investigations. However, this observation must be taken with caution due to limitation of sampling size of convict cichlids in some of these localities. However, this might constitute a gain of a new parasite after invasion, although additional samples would be required to confirm this hypothesis. Introduced species might be released from their natural enemies and therefore perform better in the new environment^59^. In addition, after colonization of a new environment invaders might acquire generalist parasites from other hosts occurring in the same area. This pattern has been mentioned in other freshwater habitats where the convict cichlid has been introduced. For instance, several species of helminth parasites transmitted by birds infect convict cichlids introduced in a Mexican river^60^. The African tilapia is a cichlid introduced in Nicaraguan waters several decades ago^61^. We studied the macroparasites of this species in two Nicaraguan lakes, and found some African parasites that do not seem to have been transmitted to native cichlids (e.g., the monogenean *Cichlidogyrus sclerosus*)*.* As recently shown by Ref.^62^ the fact that exotic parasites can be co-introduced with tilapia may determine their spillover and negative impact to the native fish fauna. Furthermore, our results show that tilapias acquired native parasites such as the widespread acantocephalan *N. costarricense* and several trematodes of the genus *Saccocoelioides*.

This study provides for the first-time records of three invasive species of parasites which have successfully spread in the Nicaraguan lakes. As mentioned before, we found the African monogenean *C. sclerosus*, but only in the African tilapia, and not yet on a native cichlid. This parasite has spread around the world together with farmed tilapia^62–65^. Tilapias were introduced in several Nicaraguan crater lakes and in both great lakes since the 50’s^61^. The other two invasive species were found in native fish, the Asian tapeworm *Schyzocotyle acheilognathi*, and the cosmopolitan anchorworm *Lernaea cyprinacea*. These two parasites are commonly introduced into freshwater systems across the globe along with cyprinids (carps) (see^66–69^). In the Americas, the Asian tapeworm had been so far only reported in two cichlid species in two tributaries of the Chagres River Panama, *Aequidens coeruleopunctatus* and *Cryptoheros paramensis*^70^. This parasite was likely introduced into the Panama Canal area with the stocking of grass carps *Ctenopharyngodon idella* for the control of aquatic vegetation. Although no carp introduction has been reported in the Nicaraguan lakes, the Asian tapeworm was already reported in Nicaraguan poeciliids^67^. However, Ref.^67^ showed that the Asian tapeworm is also commonly found in poeciliids, and they can represent the source of the infection in an alternate way. The cosmopolitan anchorworm *Lernaea cyprinacea*, is another parasite species usually co-introduced with carps; however, since no carps are known to have been introduced in Nicaraguan lakes, another source of infection must exist in other host group, but this has not yet been reported. The oribatid mite in the body cavity of *Parachromis* spp. in crater Lake Xiloá may not represent a case of invasive species. Mites are considered unusual fish parasites, although under certain environmental conditions some have been shown to proliferate and colonize weak or stressed fish^71^. Indeed, very few findings of oribatid mites have been reported (but see^72^).

Parasites are a useful independent source of information about the evolutionary history of hosts and their diversification patterns. Parasites may act as biological tags of hosts taxonomy and biogeography^52,73,74^. Additional layers of information can be gained by contrasting the genetic structure of parasites and hosts^75,76^. In African cichlids monogenean ectoparasites have been used for evaluating their contribution to the fish species diversity^52,77^. There are interesting open questions in cichlid biogeography, such as the extent of marine dispersal after Gondwanan break-up^78^, or the colonization of Central America from South American stocks^79^, and parasites might shed additional light^25,52,80^.

Over 400 species of parasites have been recorded worldwide from cichlids, and about half of them correspond to American cichlids^25^. This is considered a great underestimation of the real diversity. Increasing work is revealing hidden diversity, and even new genera are being described^81–83^. The incorporation of genetic data to evolutionary parasitology has fueled the recognition of new taxa, revealing cryptic species^84–87^, and contributing to the establishment of robust species boundaries, increasing taxonomic resolution. Most of the parasite fauna reported in this study has been found in cichlids in other areas of Middle America. This indicates a high degree of host specificity towards this group of fish. However, it seems that Lower Central America (LCA) (a region comprising Panama, Costa Rica, and Nicaragua) represents an area of high diversification. Even though parasite species as the trematode *O. manteri*, originally reported from Lake Nicaraguan cichlids^27^, reach their northern distribution limit in southeastern Mexico following the evolutionary and biogeographical history of their hosts^50^, where they experienced a diversification event^56^. However, for some genera, i.e., *Procamallanus*, *Crassicutis*, and *Neoechinorhynchus*, LCA represents the region where independent evolutionary lineages diversified^37,56,86^. Interestingly, our study did not detect instances of diversification when considering isolation of cichlids and their parasite fauna in crater lakes of Nicaragua. The parasites of freshwater fishes in Central America are still poorly known and many cichlid species remain to be studied. Comprehensive studies such as the present work provide a solid ground to further explore the evolutionary history of hosts and parasites.

## Conclusion

We have performed a comprehensive inventory of macroparasites infecting cichlid fish in Nicaraguan lakes, focusing on the Midas cichlid which represents a model system of recent repeated adaptive radiations in several crater lakes. Moreover, by including a detailed morphological and molecular analyses, this study expands our knowledge of parasite communities in freshwater fish of the Neotropical biogeographic region. We showed that the great Nicaraguan lakes hold larger parasite diversity than the smaller and younger crater lakes. However, fish populations within the large lakes analysed individually did not have more diverse parasite assemblages in comparison with those of the crater lakes. This study provides the ground for investigating host-parasite dynamics in this promising system, and the contribution of parasites to the species richness of Nicaraguan cichlids.

## Supplementary Information

Supplementary Information.

## References

[1] Price PW (1980). Evolutionary Biology of Parasites.

[2] Lima LB, Bellay S, Giacomini HC, Isaac A, Lima-Junior DP (2016). Influence of host diet and phylogeny on parasite sharing by fish in a diverse tropical floodplain. Parasitology.

[3] Eizaguirre C, Lenz TL, Kalbe M, Milinski M (2012). Rapid and adaptive evolution of MHC genes under parasite selection in experimental vertebrate populations. Nat. Commun..

[4] Bashey F (2015). Within-host competitive interactions as a mechanism for the maintenance of parasite diversity. Philos. Trans. R. Soc. B Biol. Sci..

[5] Jolles JW, Mazué GPF, Davidson J, Behrmann-Godel J, Couzin ID (2020). *Schistocephalus* parasite infection alters sticklebacks’ movement ability and thereby shapes social interactions. Sci. Rep..

[6] Demandt N (2018). Parasite-infected sticklebacks increase the risk-taking behaviour of uninfected group members. Proc. R. Soc. B Biol. Sci..

[7] Poulin R (2010). Parasite manipulation of host behavior: An update and frequently asked questions. Adv. Study Behav..

[8] Terui A, Ooue K, Urabe H, Nakamura F (2017). Parasite infection induces size-dependent host dispersal: Consequences for parasite persistence. Proc. R. Soc. B Biol. Sci..

[9] Raeymaekers JAM (2013). Contrasting parasite communities among allopatric colour morphs of the Lake Tanganyika cichlid Tropheus. BMC Evol. Biol..

[10] Meyer BS (2019). An exploration of the links between parasites, trophic ecology, morphology, and immunogenetics in the Lake Tanganyika cichlid radiation. Hydrobiologia.

[11] Gobbin TP (2020). Temporally consistent species differences in parasite infection but no evidence for rapid parasite-mediated speciation in Lake Victoria cichlid fish. J. Evol. Biol..

[12] Karvonen A, Wagner CE, Selz OM, Seehausen O (2018). Divergent parasite infections in sympatric cichlid species in Lake Victoria. J. Evol. Biol..

[13] Bush SE (2019). Host defense triggers rapid adaptive radiation in experimentally evolving parasites. Evol. Lett..

[14] Waid RM, Raesly RL, Mckaye KR, McCrary J (1999). Zoogeografía íctica de lagunas cratéricas de Nicaragua. Encuentro.

[15] Barluenga M, Stölting K, Salzburger W, Muschick M, Meyer A (2006). Sympatric speciation in Nicaraguan crater lake cichlid fish. Nature.

[16] Elmer KR, Lehtonen TK, Fan S, Meyer A (2012). Crater lake colonization by neotropical cichlid fishes. Evolution.

[17] Kautt AF (2020). Contrasting signatures of genomic divergence during sympatric speciation. Nature.

[18] Elmer KR, Lehtonen TK, Kautt AF, Harrod C, Meyer A (2010). Rapid sympatric ecological differentiation of crater lake cichlid fishes within historic times. BMC Biol..

[19] Kautt AF, Machado-Schiaffino G, Torres-Dowdall J, Meyer A (2016). Incipient sympatric speciation in Midas cichlid fish from the youngest and one of the smallest crater lakes in Nicaragua due to differential use of the benthic and limnetic habitats?. Ecol. Evol..

[20] Barluenga M, Meyer A (2010). Phylogeography, colonization and population history of the Midas cichlid species complex (*Amphilophus* spp.) in the Nicaraguan crater lakes. BMC Evol. Biol..

[21] Elmer KR, Lehtonen TK, Meyer A (2009). Color assortative mating contributes to sympatric divergence of neotropical cichlid fish. Evolution.

[22] Kautt AF, Machado-Schiaffino G, Meyer A (2018). Lessons from a natural experiment: Allopatric morphological divergence and sympatric diversification in the Midas cichlid species complex are largely influenced by ecology in a deterministic way. Evol. Lett..

[23] Elmer KR, Kusche H, Lehtonen TK, Meyer A (2010). Local variation and parallel evolution: Morphological and genetic diversity across a species complex of neotropical crater lake cichlid fishes. Philos. Trans. R. Soc. B Biol. Sci..

[24] Elmer KR (2014). Parallel evolution of Nicaraguan crater lake cichlid fishes via non-parallel routes. Nat. Commun..

[25] Vanhove MPM (2016). Cichlids: A host of opportunities for evolutionary parasitology. Trends Parasitol..

[26] Choudhury A (2016). Trematode diversity in freshwater fishes of the Globe II: ‘New World’. Syst. Parasitol..

[27] Watson DE, Thorson TB (1976). Digenea of fishes from Lake Nicaragua. Investigations of the Ichthyofauna of Nicaraguan Lakes.

[28] Aguirre-Macedo ML (2001). Larval helminths parasitizing freshwater fishes from the Atlantic coast of Nicaragua. Comp. Parasitol..

[29] Aguirre-Macedo ML (2001). Some adult endohelminths parasitizing freshwater fishes from the Atlantic Drainages of Nicaragua. Comp. Parasitol..

[30] Mendoza-Franco EF, Posel P, Dumailo S (2003). Monogeneans (Dactylogyridae: Ancyrocephalinae) of freshwater fishes from the Caribbean coast of Nicaragua. Comp. Parasitol..

[31] Andrade-Gómez L, Pinacho-Pinacho CD, García-Varela M (2017). Molecular, morphological, and ecological data of *Saccocoelioides* Szidat, 1954 (Digenea: Haploporidae) from Middle America supported the reallocation from *Culuwiya cichlidorum* to *Saccocoelioides*. J. Parasitol..

[32] López-Jiménez A, Pérez-Ponce de León G, García-Varela M (2018). Molecular data reveal high diversity of *Uvulifer* (Trematoda: Diplostomidae) in Middle America, with the description of a new species. J. Helminthol..

[33] Vidal-Martínez VM, Scholz T, Aguirre-Macedo ML (2001). Dactylogyridae of cichlid fishes from Nicaragua, Central America, with descriptions of *Gussevia herotilapiae* sp. n. and three new species of *Sciadicleithrum* (Monogenea: Ancyrocephalinae). Comp. Parasitol..

[34] de Chambrier A, Vaucher C (1984). *Proteocephalus gaspari* n. sp. (Cestoda: Proteocephalidae), parasite de *Lepisosteus tropicus* (Gill.) au Lac Managua (Nicaragua). Rev. suisse Zool..

[35] González-Solís AD, Jiménez-García MI (2006). Parasitic nematodes of freshwater fishes from two nicaraguan crater lakes. Comp. Parasitol..

[36] Santacruz A, Morales-Serna FN, Leal-Cardín M, Barluenga M, Pérez-Ponce de León G (2020). *Acusicola margulisae* n. sp. (Copepoda: Ergasilidae) from freshwater fishes in a Nicaraguan crater lake based on morphological and molecular evidence. Syst. Parasitol..

[37] Santacruz A, Barluenga M, Pérez-Ponce de León G (2021). Taxonomic assessment of the genus *Procamallanus* (Nematoda) in Middle American cichlids (Osteichthyes) with molecular data, and the description of a new species from Nicaragua and Costa Rica. Parasitol. Res..

[38] Bush AO, Lafferty KD, Lotz JM, Shostak AW (1997). Parasitology meets ecology on its own terms: Margolis et al. revisited. J. Parasitol..

[39] Rózsa L, Reiczigel J, Majoros G (2000). Quantifying parasites in samples of hosts. J. Parasitol..

[40] Krebs CJ, Krebs CJ (2014). Species diversity measures. Ecological Methodology.

[41] Dixon P (2003). VEGAN, a package of R functions for community ecology. J. Veg. Sci..

[42] R Core Team. A language and environment for statistical computing. *R Found. Stat. Comput.* (2018). https://www.R-project.org.

[43] Wickham H (2008). Elegant Graphics for Data Analysis: ggplot2.

[44] Hsieh TC, Ma KH, Chao A (2016). iNEXT-package: Interpolation and extrapolation for species diversity. Methods Ecol. Evol..

[45] Chao A (2014). Rarefaction and extrapolation with Hill numbers: A framework for sampling and estimation in species diversity studies. Ecol. Monogr..

[46] Poulin R (2014). Parasite biodiversity revisited: Frontiers and constraints. Int. J. Parasitol..

[47] Salzburger W (2018). Understanding explosive diversification through cichlid fish genomics. Nat. Rev. Genet..

[48] Barluenga M, Meyer A (2004). The Midas cichlid species complex: Incipient sympatric speciation in Nicaraguan cichlid fishes?. Mol. Ecol..

[49] Elmer KR, Meyer A (2011). Adaptation in the age of ecological genomics: Insights from parallelism and convergence. Trends Ecol. Evol..

[50] Pérez-Ponce de León G, Choudhury A (2005). Biogeography of helminth parasites of freshwater fishes in Mexico: The search for patterns and processes. J. Biogeogr..

[51] Blais J (2007). MHC adaptive divergence between closely related and sympatric African cichlids. PLoS ONE.

[52] Pariselle A (2011). The monogenean parasite fauna of cichlids: A potential tool for host biogeography. Int. J. Evol. Biol..

[53] Aguilar-Aguilar R, Salgado-Maldonado G, Contreras-Medina R, Martínez-Aquino A (2008). Richness and endemism of helminth parasites of freshwater fishes in Mexico. Biol. J. Linn. Soc..

[54] Dogiel VA, Dogiel VA, Petrushevski GK, Polyanski Y (1961). Ecology of parasites of freshwater fish. Parasitology of Fishes.

[55] Poulin R, Valtonen ET (2002). The predictability of helminth community structure in space: A comparison of fish populations from adjacent lakes. Int. J. Parasitol..

[56] Razo-Mendivil U, Rosas-Valdez R, Pérez-Ponce de León G (2009). A new Cryptogonimid (Digenea) from the mayan cichlid, Cichlasoma urophthalmus (Osteichthyes: Cichlidae), in several localities of the Yucatán Peninsula, Mexico. J. Parasitol..

[57] Mendoza-Franco EF (1995). Occurrence of *Sciadicleithrum mexicanum* Kritsky, Vidal-Martinez et Rodríguez-Canul, 1994 (Monogenea: Dactylogyridae) in the Cichlid *Cichlasoma urophthalmus* from a flooded quarry in Yucatan, Mexico. Mem. Inst. Oswaldo Cruz.

[58] Blasco-Costa I, Poulin R (2013). Host traits explain the genetic structure of parasites: A meta-analysis. Parasitology.

[59] Torchin ME, Lafferty KD, Dobson AP, McKenzie VJ, Kuris AM (2003). Introduced species and their missing parasites. Nature.

[60] Salgado-Maldonado G (2001). Helminth parasites of freshwater fishes of the Balsas River drainage basin of southwestern Mexico. Comp. Parasitol..

[61] McCrary JK, Murphy BR, Stauffer JR, Hendrix SS (2007). Tilapia (Teleostei: Cichlidae) status in Nicaraguan natural waters. Environ. Biol. Fishes.

[62] García-Vásquez A, Pinacho-Pinacho CD, Guzmán-Valdivieso I, Calixto-Rojas M, Rubio-Godoy M (2021). Morpho-molecular characterization of *Gyrodactylus* parasites of farmed tilapia and their spillover to native fishes in Mexico. Sci. Rep..

[63] Paredes-Trujillo A, Velázquez-Abunader I, Torres-Irineo E, Romero D, Vidal-Martínez VM (2016). Geographical distribution of protozoan and metazoan parasites of farmed Nile tilapia *Oreochromis niloticus* (L.) (Perciformes: Cichlidae) in Yucatán, México. Parasit. Vectors.

[64] Zhang S (2019). Monogenean fauna of alien tilapias (Cichlidae) in south China. Parasite.

[65] Outa JO, Dos Santos QM, Avenant-Oldewage A, Jirsa F (2021). Parasite diversity of introduced fish *Lates niloticus*, *Oreochromis niloticus* and endemic *Haplochromis* spp. of Lake Victoria. Kenya. Parasitol. Res..

[66] Smit NJ, Malherbe W, Hadfield KA (2017). Alien freshwater fish parasites from South Africa: Diversity, distribution, status and the way forward. Int. J. Parasitol. Parasites Wildl..

[67] Pérez-Ponce de León G, Lagunas-Calvo O, García-Prieto L, Briosio-Aguilar R, Aguilar-Aguilar R (2018). Update on the distribution of the co-invasive *Schyzocotyle acheilognathi* (= *Bothriocephalus acheilognathi*), the Asian fish tapeworm, in freshwater fishes of Mexico. J. Helminthol..

[68] Scholz T, Šimková A, Razanabolana JR, Kuchta R (2018). The first record of the invasive Asian fish tapeworm (*Schyzocotyle acheilognathi*) from an endemic cichlid fish in Madagascar. Helminthol..

[69] Acosta A, Carvalho E, da Silva R (2013). First record of *Lernaea cyprinacea* (copepoda) in a native fish species from a Brazilian river. Neotrop. Helminthol..

[70] Choudhury A (2013). The invasive asian fish tapeworm, *Bothriocephalus acheilognathi* Yamaguti, 1934, in the chagres river/panama canal drainage, Panama. BioInvas. Rec..

[71] Schatz H, Behan-Pelletier V (2008). Global diversity of oribatids (Oribatida: Acari: Arachnida). Hydrobiologia.

[72] Choudhury A, Hoffnagle TL, Cole RA (2004). Parasites of native and nonnative fishes of the Little Colorado River, Grand Canyon, Arizona. J. Parasitol..

[73] Vanhove MPM, Scholz T, Vanhove MPM, Smit N, Jayasundera Z, Gelnar M (2018). Part 6: Evolutionary parasitology of African freshwater fishes—And its implications for the sustainable management of aquatic resources. A Guide to the Parasites of African Freshwater Fishes.

[74] Catalano SR, Whittington ID, Donnellan SC, Gillanders BM (2014). Parasites as biological tags to assess host population structure: Guidelines, recent genetic advances and comments on a holistic approach. Int. J. Parasitol. Parasites Wildl..

[75] Baldwin RE, Banks MA, Jacobson KC (2011). Integrating fish and parasite data as a holistic solution for identifying the elusive stock structure of Pacific sardines (*Sardinops sagax*). Rev. Fish Biol. Fish..

[76] Criscione CD, Blouin MS (2007). Parasite phylogeographical congruence with salmon host evolutionarily significant units: Implications for salmon conservation. Mol. Ecol..

[77] Vanhove MPM (2015). Hidden biodiversity in an ancient lake: Phylogenetic congruence between Lake Tanganyika tropheine cichlids and their monogenean flatworm parasites. Sci. Rep..

[78] Matschiner M, Böhne A, Ronco F, Salzburger W (2020). The genomic timeline of cichlid fish diversification across continents. Nat. Commun..

[79] Choudhury A, García-Varela M, Pérez-Ponce de León G (2017). Parasites of freshwater fishes and the Great American biotic interchange: A bridge too far?. J. Helminthol..

[80] Mendoza-Franco EF, Vidal-Martínez VM (2005). Phylogeny of species of *Sciadicleithrum* (Monogenoidea: Ancyrocephalinae), and their historical biogeography in the Neotropics. J. Parasitol..

[81] de Chambrier A, Pinacho-Pinacho CD, Hernández-Orts JS, Scholz TT (2017). A new genus and two new species of proteocephalidean tapeworms (Cestoda) from cichlid fish (Perciformes: Cichlidae) in the neotropics. J. Parasitol..

[82] Mendoza-Palmero CA, Blasco-Costa I, Hernández-Mena D, Pérez-Ponce de León G (2017). *Parasciadicleithrum octofasciatum* n. gen., n. sp. (Monogenoidea: Dactylogyridae), parasite of *Rocio octofasciata* (Regan) (Cichlidae: Perciformes) from Mexico characterised by morphological and molecular evidence. Parasitol. Int..

[83] Pinacho-Pinacho CD, Hernández-Orts JS, Sereno-Uribe AL, Pérez-Ponce de León G, García-Varela M (2017). *Mayarhynchus karlae* n. g., n. sp. (Acanthocephala: Neoechinorhynchidae), a parasite of cichlids (Perciformes: Cichlidae) in southeastern Mexico, with comments on the paraphyly of *Neoechynorhynchus* Stiles & Hassall, 1905. Syst. Parasitol..

[84] Razo-Mendivil U, Vázquez-Domínguez E, Rosas-Valdez R, Pérez-Ponce de León G, Nadler SA (2010). Phylogenetic analysis of nuclear and mitochondrial DNA reveals a complex of cryptic species in *Crassicutis cichlasomae* (Digenea: Apocreadiidae), a parasite of Middle-American cichlids. Int. J. Parasitol..

[85] Razo-Mendivil U, Rosas-Valdez R, Rubio-Godoy M, Pérez-Ponce de León G (2015). The use of mitochondrial and nuclear sequences in prospecting for cryptic species in *Tabascotrema verai* (Digenea: Cryptogonimidae), a parasite of *Petenia splendida* (Cichlidae) in Middle America. Parasitol. Int..

[86] Pinacho-Pinacho CD, García-Varela M, Sereno-Uribe AL, Pérez-Ponce de León G (2018). A hyper-diverse genus of acanthocephalans revealed by tree-based and non-tree-based species delimitation methods: Ten cryptic species of *Neoechinorhynchus* in Middle American freshwater fishes. Mol. Phylogenet. Evol..

[87] Martínez-Aquino A (2009). Detecting a complex of cryptic species within *Neoechinorhynchus golvani* (Acanthocephala: Neoechinorhynchidae) inferred from ITSs and LSU rDNA gene sequences. J. Parasitol..

