# Supplementary material for: The macroparasite fauna of cichlid fish from Nicaraguan lakes, a model system for understanding host–parasite diversification and speciation
Source: Sci Rep. 2022 Mar 10;12:3944. doi: 10.1038/s41598-022-07647-w (PMC8913791; doi:10.1038/s41598-022-07647-w)

Supplementary Material

The macroparasite fauna of cichlid fish from Nicaraguan lakes, a model system for understanding host-parasite diversification and speciation

Ana Santacruz, Marta Barluenga and Gerardo Pérez-Ponce de León

Supplementary Table S1. GenBank accession numbers and CNHE or CNCR catalog numbers.

Parasite	cox1	cox2	28S	18S	ITS	CNHE	CNCR
**Trematoda**							
Ascocotyle pindoramensis	−	−	OL960048−50	−	−	−	−
Austrodiplostomum compactum	−	−	−	−	−	11496	−
Crassicutis cichlasomae	OM514320−23	−	−	−	OL872180	11483−11486	−
Ithyoclinostomum yamagutii	−	−	−	−	−	11498	−
Heterophyidae gen. sp.	−	−	−	−	−	11504	−
Oligogonotylus manteri	OM514349−52	−	OL960047	−	−	11487−11490	−
Phostodiplostomum sp. 1	−	−	−	−	−	11500	−
Phostodiplostomum sp. 2	−	−	−	−	−	11501	−
Saccocoelioides orosiensis	OM540397−99	−	−	−	−	−	−
Saccocoelioides cf. *lamothei*	OM540400−01	−	−	−	−	−	−
**Monogenea**							
Sciadicleithrum mexicanum	OM540389−94	−	OL960045	−	−	11475−11480	−
Sciadicleithrum nicaraguensis	−	−	OL960043−44	−	−	11481	−
Sciadicleithrum sp. 1	OM540395	−	OL960046	−	−	−	−
**Acanthocephala**							
Neoechinorynchus costarricense	OM540396	−	OL960051	−	−	11507	−
**Nematoda**							
Contracaecum sp. 1	−	OM524389	−	−	−	−	−
Hysterothylacium sp.	−	−	−	−	−	11506	−
Physocephalus sp.	−	−	−	−	−	11505	−
Procamallanus barlowi	MW487882−87	−	MW485591−93	−	−	11472−11473	−
**Copepoda**							−
Acusicola margulisae	MN854838−70	−	MN852849−51	MN852694−96	−	−	35553−35553
Ergasilidae gen. sp.	OM541914	−	−	−	−	−	−
Lernaeidae gen. sp.	OM541913	−	−	−	−	−	−

Supplementary Table S2. Primers used for PCR and sequencing of parasites in this study.

Genetic region	Primer name	F/ R	Sequence (5'− 3')	Reference
cox1	JB3/ Asmit1	F	TTTTTTGGGCATCCTGAGGTTTAT	[1]
	JB4.5	R	TAAAGAAAGAACATAATGAAAATG	[1]
	Schisto3	R	TAATGCATMGGAAAAAAACA	[2]
	507	F	AGTTCTAATCATAARGATATYGG	[3]
	HCO2198	R	TAAACTTCAGGGTGACCAAAAAATCA	[4]
	pr-b	R	AGTTCTAATCATAARGATATYGG	[5]
	MplatCOX1dF	F	TGTAAAACGACGGCCAGTTTWCITTRGATCATAAG	[6]
	MplatCOX1dR	R	CAGGAAACAGCTATGACTGAAAYAAYAIIGGATCICCACC	[6]
cox2	210	F	TTTTCTAGTTATATAGATTGRTTYAT	[7]
	211	R	CACCAACTCTTAAAATTATC	[7]
28S	391	F	AGCGGAGGAAAAGAAACTAA	[8]
	536	R	CAGCTATCCTGAGGGAAAC	[9]
	502*	F	CAAGTACCGTGAGGGAAAGTTGC	[9]
	28SF	F	ACAACTGTGATGCCCTTAG	[10]
	28SR	R	TGGTCCGTGTTTCAAGACG	[10]
	U178	F	GCACCCGCTGAAYTTAAG	[2]
	1200R*	R	GCATAGTTCACCATCTTTCGG	[2]
	L1642	R	CCAGCGCCATCCATTTTCA	[2]
18S	18SF	F	AAGGTGTGMCCTATCAACT	[10]
	18SR	R	TTACTTCCTCTAAACGCTC	[10]

F: forward primer; R: reverse primer

Asterisk indicates primers used only in sequencing reaction.

REFERENCES

1. Bowles, J., Blair, D. & McManus, D. P. Genetic variants within the genus *Echinococcus* identified by mitochondrial DNA sequencing. *Mol. Biochem. Parasitol.* **54**, 165–173 (1992).
2. Lockyer, A. E., Olson, P. D. & Littlewood, D. T. J. Utility of complete large and small subunit rRNA genes in resolving the phylogeny of the Neodermata (Platyhelminthes): Implications and a review of the cercomer theory. *Biol. J. Linn. Soc.* **78**, 155–171 (2003).
3. Nadler, S. A., Bolotin, E. & Stock, S. P. Phylogenetic relationships of *Steinernema* Travassos, 1927 (Nematoda: Cephalobina: Steinernematidae) based on nuclear, mitochondrial and morphological data. *Syst. Parasitol.* **63**, 161–181 (2006).
4. Black, M. *et al.* DNA primers for amplification of mitochondrial cytochrome c oxidase subunit I from diverse metazoan invertebrates. *Mol. Mar. Biol. Biotechnol.* **3**, 294–299 (1994).
5. Bessho, Y., Ohama, T. & Osawa, S. Planarian mitochondria I. Heterogeneity of cytochrome c oxidase subunit I gene sequences in the freshwater planarian, *Dugesia japonica*. *J. Mol. Evol.* **34**, 324–330 (1992).
6. Moszczynska, A., Locke, S. A., McLaughlin, J. D., Marcogliese, D. J. & Crease, T. J. Development of primers for the mitochondrial cytochrome c oxidase i gene in digenetic trematodes (Platyhelminthes) illustrates he challenge of barcoding parasitic helminths. *Mol. Ecol. Resour.* **9**, 75–82 (2009).
7. Nadler, S. A. & Hudspeth, D. S. S. Phylogeny of the ascaridoidea (Nematoda: Ascaridida) based on three genes and morphology: Hypotheses of structural and sequence evolution. *J. Parasitol.* **86**, 380–393 (2000).
8. Nadler, S. A. & Hudspeth, D. S. S. Ribosomal DNA and Phylogeny of the Ascaridoidea (Nemata: Secernentea): Implications for Morphological Evolution and Classification. *Mol. Phylogenet. Evol.* **10**, 221–236 (1998).
9. García-Varela, M. & Nadler, S. A. Phylogenetic relationships of palaeacanthocephala (Acanthocephala) inferred from SSU and LSU rDNA gene sequences. *J. Parasitol.* **91**, 1401–1409 (2005).
10. Song, Y., Wang, G. T., Yao, W. J., Gao, Q. & Nie, P. Phylogeny of freshwater parasitic copepods in the Ergasilidae (Copepoda: Poecilostomatoida) based on 18S and 28S rDNA sequences. *Parasitol. Res.* **102**, 299–306 (2008).

Supplementary Table S3. Species of cichlids sampled in two great and five crater lakes of Nicaragua between 2017 and 2019, indicating overall prevalence of macroparasites per sampling year. Ne. Number of fish examined, Ni. Number of fish infected, P. prevalence (%).

Lake	Host species	Host code	Year									
			2017			2018			2019			
			Ne	Ni	P	Ne	Ni	P	Ne	Ni	P	Total
Asososca León (108)	***Amphilophus citrinellus***	AciAsL	20	20	100	28	28	100	23	23	100	71
	Oreochromis sp.	OreAsL	3	3	100	3	3	100	8	3		14
	Parachromis spp.	ParAsL	5	5	100	12	12	100	6	6	100	23
Apoyeque (26)	***Amphilophus citrinellus***	AciAye	22	22	100	–	–	–	–	–	–	22
	Parachromis spp.	ParAye	4	4	100	–	–	–	–	–	–	4
Apoyo (117)	***Amphilophus astorquii***	AasApo	16	16	100	4	4	100	7	7	100	27
	Amphilophus chancho	AchApo	15	15	100	5	5	100	13	13	100	33
	Amphilophus flavealus	AflApo	1	1	100	–	–	–	–	–	–	1
	Amphilophus zaliosus	AzaApo	14	14	100	5	5	100	14	14	100	33
	Amphilophus globosus	AglApo	–	–	–	–	–	–	3	3	100	3
	Amatitlania nigrofasciata	NigApo	–	–	–	11	10	90.9	6	5		17
	Parachromis spp.	ParApo	–	–	–	3	3	100	–	–		3
Masaya (39)	***Amphilophus citrinellus***	AciMas	25	25	100	10	10	100	–	–	–	35
	Cribroheros longimanus	LonMas	3	3	100	–	–	–	–	–	–	3
	Hypsophrys nematopus	NeeMas	–	–	–	1	1	100	–	–	–	1
Xiloá (145)	***Amphilophus amarillo***	AmaXil	16	16	100	5	3	60	1	1	100	22
	Amphilophus sagittae	AsaXil	16	16	100	27	27	100	6	6	100	49
	Amphilophus xiloaensis	AxiXil	11	11	100	4	4	100	7	7	100	22
	Amatitlania nigrofasciata	NigXil	–	–	–	5	3		4	4	100	9
	Cribroheros longimanus	LonAxil	–	–	–	2	2	100	3			5
	Cribroheros rostratus	RosXil	–	–	–	3	3	100	4	4	100	7
	Hypsophrys nematopus	NeeXil	–	–	–	4	2	50	3	2		7
	Hypsophrys nicaraguensis	HypXil	–	–	–	7	7	100	6			13
	Parachromis spp.	ParXil	5	5	100	5	5	100	1	1	100	11
Managua (152)	***Amphilophus citrinellus***	AciMan	30	29	96.6	30	30	100	26	26	100	86
	Amphilophus labiatus	AlaMan	9	9	100	7	7	100	5	5	100	21
	Cribroheros rostratus	RosMan	1	1	100	3	3	100	6	5		10
	Hypsophrys nematopus	NeeMan	–	–	–	–	–	–	8		100	8
	Oreochromis sp.	OreMan	–	–	–	6	0	0	11	7		17
	Parachromis spp.	ParMan	3	3	100	4	4	100	3	3	100	10
Nicaragua (167)	***Amphilophus citrinellus***	AciNic	9	9	100	21	21	100	15	15	100	45
	Amphilophus labiatus	AlaNic	14	14	100	13	13	100	12	12	100	39
	Amatitlania nigrofasciata	NigNic	–	–	–	–	–	–	3		100	3
	Archocentrus centrarchus	CenNic	–	–	–	–	–	–	8	7		8
	Cribroheros longimanus	LonNic	–	–	–	1	1	100	2	2	100	3
	Cribroheros rostratus	RosNic	–	–	–	8	8	100	3		100	11
	Herotilapia multispinosa	HmuNic	–	–	–	–	–	–	1	1	100	1
	Hypsophrys nematopus	NeeNic	–	–	–	13	13	100	1	1	100	14
	Hypsophrys nicaraguensis	HypNic	–	–	–	13	12		3	2		16
	Parachromis spp.	ParNic	3	3	100	6	6	100	14	14	100	23
	Vieja sp.	VieNic	1	0	0	3	3	100	–	–	–	4

Supplementary Table 4. Host-parasite records within lakes. In bold the Midas cichlids species are indicated.

Lake	Species	Host species
**Trematoda**		
Nicaragua	*Ascocotyle pindoramensis*	***A. labiatus***
	Crassicutis cichlasomae	***A. citrinellus, A. labiatus**, A. centrarchus, A. nigrofasciata, C. rostratus, H. nicaraguensis, H. nematopus, Parachromis* spp., *Vieja* sp.
	Ithyoclinostomum yamagutii	*H. nicaraguensis, H. nematopus, Parachromis* spp.
	Oligogonotylus manteri	***A. citrinellus, A. labiatus**, C. rostratus, H. nicaraguensis, H. nematopus, Parachromis* spp.
	Posthodiplostomum sp. 1	*H. nematopus*
	Posthodiplostomum sp. 2	*A. nigrofasciata*
	Saccocoelioides cf. *lamothei*	*H. nematopus*
	Saccocoleioides spp.	*H. nematopus*
	Strigeidae gen sp.	*H. nematopus*
Managua	*Ascocotyle pindoramensis*	*Parachromis sp.*
	Austrodiplostomum compactum	***A.citrinellus**, C. rostratus, Parachromis* spp.
	Crassicutis cichlasomae	***A. citrinellus, A. labiatus**, C. rostratus, Parachromis* spp.
	Cryptogonomidae gen. sp.	*Parachromis spp.*
	Heterophyidae gen. sp.	*Oreochromis* sp.
	Oligogonotylus manteri	***A. citrinellus, A. labiatus**, C. rostratus, H. nematopus, Parachromis* spp.
	Saccocoleioides spp.	***A. citrinellus**, Oreochromis* sp., *Parachromis* spp.
Asososca León	*Crassicutis cichlasomae*	***A. citrinellus***
	Oligogonotylus manteri	***A. citrinellus,** Parachromis* spp.
	Saccocoelioides cf. *lamothei*	***A. citrinellus**, Oreochromis* sp.
	Saccocoelioides orosiensis	*Oreochromis* sp.
	Saccocoleioides spp.	***A. citrinellus**, Oreochromis* sp.
Apoyeque	*Crassicutis cichlasomae*	***A. citrinellus**, Parachromis* spp.
	Oligogonotylus manteri	***A. citrinellus**, Parachromis* spp.
Xiloá	*Crassicutis cichlasomae*	***A. amarillo, A. sagittae, A. xiloaensis**, C. longimanus, A. nigrofasciata, C. rostratus, H. nicaraguensis*
	Oligogonotylus manteri	***A. amarillo, A. sagittae, A. xiloaensis**, C. longimanus, C. rostratus, H. nicaraguensis, H. nematopus, Parachromis* spp.
	Saccocoelioides orosiensis	***A. sagittae, A. xiloaensis**, H. nicaraguensis, H. nematopus*
	Saccocoleioides spp.	***A. sagittae***
Masaya	*Crassicutis cichlasomae*	***A. citrinellus**, C. longimanus*
	Oligogonotylus manteri	***A. citrinellus**, H. nematopus*
	Posthodiplostomum sp. 1	*H. nematopus*
Apoyo	*Crassicutis cichlasomae*	***A. astorquii, A. chancho, A. globosus, A. flaveolus, A. zaliosus**, Parachromis* spp.
	Oligogonotylus manteri	***A. astorquii, A. chancho, A. globosus, A. zaliosus**, Parachromis spp.*
	Saccocoelioides orosiensis	***A. astorquii, A. chancho, A. zaliosus**, A. nigrofasciata*
	Saccocoleioides spp.	***A. astorquii, A. chancho, A. flaveolus, A. globosus, A. zaliosus***

**Cestoda**		
Nicaragua	*Cichlidocestus janikae*	***A. citrinellus***, *H. nicaraguensis*
Managua	*Schyzocotyle acheilognathi*	***A. citrinellus***
Xiloá	*Schyzocotyle acheilognathi*	*Parachromis* spp.
**Monogenea**		
Nicaragua	*Sciadicleithrum mexicanum*	***A. citrinellus***, *Parachromis* spp.
	Sciadicleithrum sp. 2	*C. rostratus*
Managua	*Sciadicleithrum mexicanum*	***A. citrinellus***, *Parachromis* spp.
Asososca León	*Cichlidogyrus sclerosus*	*Oreochromis* sp.
	Sciadicleithrum mexicanum	*Parachromis* spp.
Apoyeque	*Sciadicleithrum mexicanum*	*Parachromis* spp.
Xiloá	*Sciadicleithrum mexicanum*	***A. amarillo, A. sagittae, A. xiloaensis***
	Sciadicleithrum sp. 1	*A. nigrofasciata*
Masaya	*Sciadicleithrum mexicanum*	***A. citrinellus***
	Sciadicleithrum nicaraguensis	*C. longimanus*
Apoyo	*Sciadicleithrum mexicanum*	***A. astorquii, A. flaveolus, A. chancho, A. zaliosus***, *Parachromis* spp.
**Acantocephala**		
Nicaragua	*Polymorphus brevis*	***A. citrinellus, A. labiatus***, *H. nematopus, Parachomis spp.,Vieja* sp.
	Neoechinorynchus costarricense	***A. labiatus***, *A. nigrofasciata, H. nematopus, H. nicaraguensis, H. multispinosa, Parachromis* spp.
Managua	*Neoechinorynchus costarricense*	***A. citrinellus***
Asososca León	*Neoechinorynchus costarricense*	***A. citrinellus***, *Parachromis* spp., *Oreochromis* sp.
Apoyeque	*Neoechinorynchus costarricense*	***A. citrinellus***, *Parachromis* spp.
Xiloá	*Neoechinorynchus costarricense*	***A. amarillo, A. xiloaensis, A. sagittae,*** *A. nigrofasciata, C. longimanus, Parachromis* spp.
Masaya	*Neoechinorynchus costarricense*	***A. citrinellus***
Apoyo	*Neoechinorynchus costarricense*	***A. astorquii, A. chancho, A. zaliosus***, *A. nigrofasciata, Parachromis* spp.
**Nematoda**		
Nicaragua	*Contracaecum* sp. 1	***A. citrinellus, A. labiatus***, *A. centrarchus, C. rostratus, H. nicaraguensis, H. nematopus, Parachromis* spp., *Vieja* sp.
	Physocephalus sp.	***A. labiatus***
	Procamallanus barlowi	***A. citrinellus***, ***A. labiatus***, *C. longimanus, C. rostratus, H. nicaraguensis, H. nematopus*
	Rhabdochona sp.	***A. citrinellus***
Managua	*Contracaecum* sp. 1	***A. citrinellus, A. labiatus***, *C. rostratus, Parachromis* spp.
	Procamallanus barlowi	***A. citrinellus, A. labiatus***, *Parachromis* spp.,
Asososca León	*Contracaecum* sp. 1	***A. citrinellus***, *Parachromis* spp.
	Contracaecum sp. 2	***A. citrinellus***, *Parachromis* spp.
	Contracaecum sp. 3	***A. citrinellus***, *Parachromis* spp.
	Procamallanus barlowi	***A. citrinellus***
Apoyeque	*Procamallanus barlowi*	*Parachromis* spp.
Xiloá	*Contracaecum* sp. 1	***A. amarillo, A. sagittae, A. xiloaensis***, *C. rostratus, H. nicaraguensis, H. nematopus, Parachromis* spp.
	Goezia sp.	***A. sagittae***

	Hysterothylacium sp.	*Parachromis* spp.
Masaya	*Contracaecum* sp. 1	***A. citrinellus***, *C. longimanus*
	Procamallanus barlowi	***A. citrinellus***, *C. longimanus*
Apoyo	*Contracaecum* sp. 1	***A. astorquii, A. chancho, A. zaliosus,*** *Parachromis* spp.
	Goezia sp.	***A. zaliosus***
	Procamallanus barlowi	***A. astorquii, A. chancho, A. zaliosus***, *A. nigrofasciata, Parachromis* spp.
**Hirudinea**		
Nicaragua	*Myzobdella* sp.	*Parachromis* spp.
**Copepoda**		
Nicaragua	*Acusicola* sp.	***A. citrinellus, A. labiatus***
	Ergasilidae gen. sp.	***A. citrinellus, A. labiatus***, *H. nematopus, H. nicaraguensis, Parachromis* spp., *Vieja* sp.
Managua	Ergasilidae gen. sp.	***A. citrinellus, A. labiatus***, *C. rostratus, Parachromis* spp.
	Lernaea cyprinacea	*H. nematopus*
	Lernaeidae gen. sp.	***A. citrinellus, A. labiatus***
Asososca León	*Acusicola margulisae*	***A. citrinellus***, *Parachromis* spp., *Oreochromis* sp.
Xiloá	Ergasilidae gen. sp.	***A. amarillo, A. sagittae, A. xiloaensis,*** *A. nigrofasciata, C. longimanus, H. nicaraguensis, Parachromis* spp.
Apoyo	Ergasilidae gen. sp.	***A. astorquii***
**Branchiura**		
Asososca León	*Argulus* sp.	***A. citrinellus***
Xiloá	*Argulus* sp.	***A. sagittae***
**Acariformes**		
Xiloá	Oribatide gen. sp.	*Parachromis* spp.

Supplementary Fig. S1. Matrix of mean parasite intensity per host and lake. Dark colors represent increasing intensity. White cells indicate absences. Host codes are according to Supplementary Table S3. 1) *Contracaecum* spp. and *Hystherothylacium* sp., 2) *S. orosiensis* and *S. cf. lamothei,* and 3) Ergasilidae copepods, were condensed each into a single taxon, and their mean intensities were calculated together.

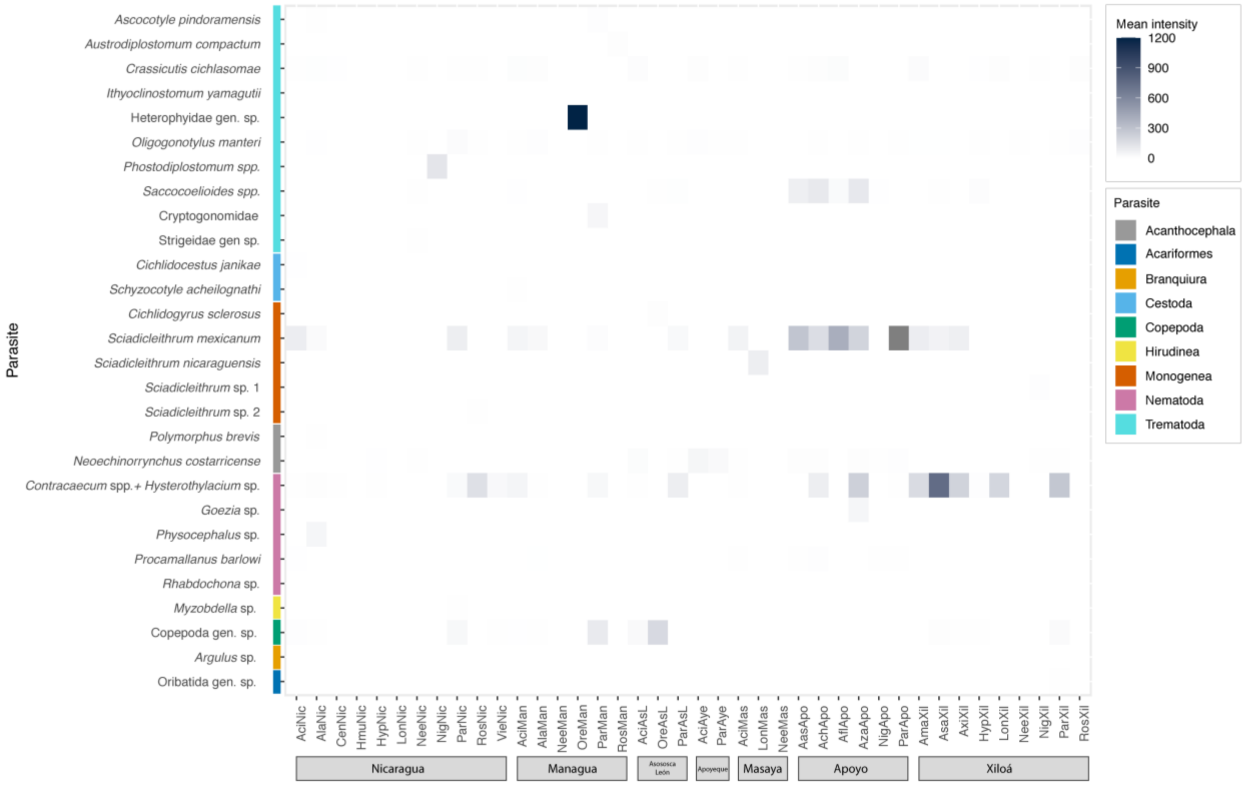

Supplement: Supplementary file 1 — Supplementary Information. [file 41598_2022_7647_MOESM1_ESM.pdf]
